# Correspondence

**Published:** 1873-02

**Authors:** Charles S. Lewis

**Affiliations:** Baltimore


### CORRESPONDENCE.

BALTIMORE, October 12, 1872.

*Editors Southern Medical Record:*

Being in the great city of Baltimore, with several leisure days on my hands, I have devoted them to visiting the medical schools now in operation here, which commenced their courses of lectures during the first days of the present month. There are now three schools of medicine in Baltimore, and I should think the first impression made upon the mind of any intelligent physician would be one of painful surprise that schools of medicine should be so multiplied. The remark is not intended to apply more to Baltimore than to the whole country, since every State (with the exception perhaps of half a dozen) has from one to three or four of these institutions within their borders, amounting, in the aggregate, to forty or fifty schools. In view of this state of things, the question naturally arises, What good is to be gained, what end attained, by so large a number of schools, with, in many instances, almost empty halls and lecture-rooms to echo to the voices of their professors? It is fair to presume that the general welfare of the profession, as well as high educational attainments, are the prime ends proposed by thus augmenting the number of schools, and that their springing up side by side would seem to argue that an active competition is absolutely necessary to the proper teaching of medicine. If this was true, it would do; but it is proven in numerous cases to be just the reverse of the truth. It is an axiom in political economy that there should be some relation of equality between "supply and demand" for a wholesome state of trade, and as it is much to be feared that medical institutions have, in many instances, become matters of trade, rather than what they purport to be—trading in other things, rather than subserving the true interests of education—it is to be lamented that the school supply should so far exceed the demand for it. We all know that this state of things has not, at any time, fostered a healthy state of medical education in the United States. Of the many evils that attend upon this much vexed subject, probably this is one of the greatest it has to contend with. There is no well-informed medical man who does not know and deplore the various artifices now practiced by many schools to attract pupils, and some of them seem determined to fill their halls at any sacrifice, even if they have to descend to the wretched system of eleemosynary education for the largest part of their students. This miserable rivalry—this trading in educational interests—is, in a great measure, the offspring of an undue and unnecessary number of schools—all competing for pupils; all eager to receive them, no matter what their moral status or preliminary education—so that, for such a mass of ignorance, it is absolutely necessary that the standard for the degree of M. D. should be low. The consequences of this state of things are seen from one end of the land to the other, and the banners of the physician are trailing in the dust as far as the evil extends. So far as I know, the professional chairs of most of the schools are filled by men of ability, and competent to teach; but when they come to confer the degree of M. D., (with a few honorable exceptions), they have to do so with a loose hand, or their pupils flee away to other and more facile institutions, there being too many such ready to receive them with open arms.

It gives me pleasure to say that the professional chairs in the schools of Baltimore are not only ably filled, but they are held by men of rank as high as any in this country. They are not only highly educated and accomplished, but many of them are truly learned men. The old Maryland University still holds up her head, and is much in advance of her rivals in the number of her pupils. Her professors are all men of first reliability. I have never heard a superior lecturer to Dr. Miles, and very much doubt if his equal is to be found in the United States on his branch (anatomy.) He is a host within himself, and would, I should think, alone, attract numerous pupils to this school. Dr. Chanciller, of the Washington school, is also a very accomplished man, and I should judge, from his appearance, that he is a high-hearted and genial gentleman and companion. Dr. Warren, of the school of "Surgeons and Physicians," is said to be (I did not have the pleasure of hearing him, although I attended at his usual hour for the purpose), one of the leading celebrities of his school, and is, I am informed, a highly finished and popular lecturer and teacher.

The material for clinical instruction, I am informed, is abundant, and students will find in Baltimore as much in this line as they will find elsewhere in any of the schools in this country. I have only to say, if students *will go away* from their own State institutions, I can see no reason for their going farther *North* than Baltimore; for they will find there every facility, every means and opportunity that they can find anywhere for being taught medicine—with active, able and "live men," fully and thoroughly informed up to the times, and, indeed, many of them in advance of them, as teachers. I regret I have been obliged to make the number of schools in Baltimore the suggestive idea for speaking my sentiments as to the needless number of schools there and elsewhere throughout the country.

I come now to speak of some of the specialties practiced in Baltimore. There are several eye and ear infirmiries or institutes, and perhaps the same number for treating diseases of the throat and chest, in the city. It was to obtain advice and aid for a diseased eye that took me to Baltimore. Fortunately for me, I fell into the hands of Dr. George Reuling, the Chief Surgeon of the Maryland Eye and Ear Institute—than whom, in my opinion, there is not a more accomplished oculist on this continent. It gives me pleasure, not only to bear witness to his skill in the treatment of diseases of the eye, and also to his almost wonderful dexterity in performing all the delicate surgical operations upon that organ, but I am constrained to express my most grateful remembrance of his kindness, tenderness and attention, accompanied by the most urbane, polished and genial manners—with a tact in this respect which makes it appear that, instead of conferring favor on you, he was rather receiving it at your hands; and that this was the genuine out-come of the heart, was proven by the fact that the same was extended to the pauper in his rags, as well as to him who came to him clothed in "purple and fine linen," and numbers of both classes were to be seen daily in his rooms. It is also my wish to make my acknowledgments for the kindness and attention I received from the talented and gentlemanly Assistant Surgeon of the Institute, Dr. Munikheysen. There are other gentlemen in the city engaged in the same line of practice, and stand deservedly high. I make mention of this fact in order to prevent any imputation or appearance of attempting to give one man a preëminence over another. Dr. Chisholm and Dr. Reuling stand upon too high a level to be affected by the opinion of any man, or to be reached by the petty rivalries, jealousies and cavils that disfigure, degrade and disgrace those who indulge in them. I mention Dr. Chisolm because it is impossible to go to Baltimore, as I did, for the treatment of a diseased eye, and not hear of him; and I speak of him from authority in which I have every confidence.

CHARLES S. LEWIS, M.D.

